# Do We Pay Enough Attention to Culture Conditions in Context of Perinatal Outcome after In Vitro Fertilization? Up-to-Date Literature Review

**DOI:** 10.1155/2016/3285179

**Published:** 2016-01-28

**Authors:** Piotr Marianowski, Filip A. Dąbrowski, Aleksandra Zyguła, Mirosław Wielgoś, Iwona Szymusik

**Affiliations:** 1st Department of Obstetrics and Gynecology, Medical University of Warsaw, Starynkiewicza Square 1/3, 02-015 Warsaw, Poland

## Abstract

Adverse perinatal outcomes in singleton IVF pregnancies have been most often explained by parental underlying diseases and so far laboratory conditions during embryo culture are still not explored well. The following review discusses the current state of knowledge on the influence of IVF laboratory procedures on the possible perinatal outcome. The role of improved media for human embryo culture is unquestionable. Addition of certain components to culture media and their effect on embryo survival and implantation rates have been taken into consideration recently and studied on animal model. Impact of media on perinatal outcome in IVF offspring has also been studied. It has been discovered that epigenetic changes and neonatal birth weight are probably associated with the use of specific culture media, as is the relation between placental size and its influence on perinatal outcome. There are still questions in the discussion about duration of embryo culture (cleavage stage versus blastocyst transfer). Some of the IVF methods, such as in vitro maturation of oocytes and freezing/thawing procedures, also require well-powered randomized controlled trials in order to define their exact impact on perinatal outcome. Constant further research is needed to assess the impact of laboratory environment on fetal and postnatal development.

## 1. Introduction

After over 30 years of existence, in vitro fertilization (IVF) is now considered a routine medical practice in the management of infertility. Deliveries following IVF account for constantly increasing percentage of all births. According to 2012 estimates the number of children born after assisted reproduction techniques exceeded 4 million. Available national birth cohorts show that the proportion of IVF infants range from 0.8 to 4.1% of all deliveries [1]. In the United States approximately 1.5% of all births and 20% of all multiple births are the result of IVF, while in Belgium and Denmark infants conceived by assisted reproduction technology (ART) comprise 3.5 percent of births [2].

Potential adverse perinatal outcomes in singleton IVF pregnancies such as early loss, spontaneous abortion, ectopic pregnancy, preterm birth, low birth weight, and being small for gestational age (SGA) have been studied in depth and are well known. In vitro fertilization has been associated with an increased risk of pregnancy complications such as placenta praevia and placental abruption, gestational hypertension, preeclampsia, and cesarean delivery [3]. However, the absolute increase in risk has generally been small and most such pregnancies have normal outcomes.

Most of the abovementioned complications may be explained by maternal and paternal underlying medical conditions associated with subfertility and infertility such as sperm factors, the use of fertility medications, laboratory conditions during embryo culture, culture media, cryopreservation and thawing, prenatal genetic diagnosis (if performed), differences in obstetrical management, increased proportion of multiple gestations and vanishing twins, or a combination of these factors [4, 5].

Until now clinical practice has shown that the in vitro culture of human embryos does not confer major adverse effects on the offspring, but possible consequences in late childhood or adulthood are still to be explored, as even the eldest children conceived by ART are still quite young.

The presented literature review is focused on the impact of embryo culture conditions on perinatal outcome after in vitro fertilization procedures. Alongside infertility causes, the factors related to culture conditions seem to be the most difficult to explore.

## 2. The Role of Embryo Culture Media: How Can They Affect Embryo Developmental Potential?

It is obvious that the quality of an embryo is one of the main factors, if not the most important, affecting the cumulative pregnancy rates after in vitro fertilization treatment, especially when elective single embryo transfer (eSET) policy is applied. In order to be a successful IVF center with eSET policy, having a highly efficient embryo freezing program is a must. Nordic countries are European leaders of the abovementioned approach, reaching 51.3% cumulative live birth rates per oocyte pickup [6]. It is very difficult however to show how embryo quality affects maternal or neonatal outcome. There are few studies demonstrating the obvious that the transfer of poor quality embryo results in a higher rate of miscarriage and lower ongoing pregnancy rates. The same studies however show that if a pregnancy continues to term, the rate of further perinatal complications is similar to good quality embryos, suggesting that poor embryo quality does not necessarily affect adverse perinatal outcome [7, 8].

There would never be any success of human IVF if it was not for the efficient laboratory techniques. The technical and scientific progress, together with the proper quality control in the IVF laboratory, has an undisputed role in maintaining satisfactory clinical results. Alongside technical aspects, the role of improved media for human embryo culture is unquestionable. Extensive knowledge regarding the composition of culture media used in human IVF comes from animal studies. It has been well documented that, for example, certain amino acids are integral components of embryo culture media, as they are important at all stages of development and serve as energy source needed to maintain homeostasis [9]. Nevertheless, they break down spontaneously and release ammonia into the culture media. It has been proven that the accumulation of ammonia has a negative impact on embryo physiology, viability, and fetal development in mice. Biggers et al. demonstrated that the excessive amount of amino acids (or an extended culture without media renewal) may cause neural tube defects, such as exencephaly in mice. Moreover, mouse embryo development was slower in the accumulated ammonia after 48 hours of culture; the rate of compaction and blastocyst formation was also significantly lower [10]. Therefore, their study raised a question regarding the most appropriate concentration of amino acids. It is also known that human embryos show delayed development on day 3 and reduced rate of blastocyst formation when cultured in the presence of increased concentration of amino acids. Embryo metabolism is also affected, but—which is interesting—not from day 3 onwards. There are different demands and requirements in pre- and postcompaction stages [9]. Since ammonia is detrimental to embryo development, the majority of commercially available culture media should be renewed at least every 48 hours (sequential media are therefore preferred in the IVF lab over monoculture). Ammonia buildup is also thought to be responsible for the reduced ability to implant and increased rate of fetal loss if pregnancy is achieved [11]. Based on animal studies, it seems that the cleavage stage embryo is more vulnerable and sensitive to in vivo induced stressors and most dependent on ionic and pH conditions. It is also worth remembering that the composition of media may influence the alteration of gene expression (especially lack of amino acids)—it was shown that methylation patterns can be changed even in the two-cell embryo. It could be the explanation for an increased risk of imprinting disorders in IVF neonates (such as Beckwith-Wiedemann or Angelman syndrome) [11]. Most recently Wale and Gardner published an extensive review on the effects of chemical and physical factors on mammalian embryo culture. They analyzed the effect of oxygen concentration, ammonia concentration, volatile organics in poor laboratory air quality, the source of albumin in media, the optimal temperature and pH, the use of oil overlay, and incubation liquid volume and the role of light and stress induced by pipetting. Their vast analysis of mostly mammalian studies shows that a combination of all the above can influence the viability of human embryos and the long-term effect on the fetus [12]. It seems that the optimal embryo development conditions are still to be achieved, as currently used systems are not yet perfect and still encounter some form of compromise.

Some authors also studied the addition of certain components to culture media and their effect on embryo survival and implantation rates. Ziebe et al. analyzed the effect of addition of granulocyte-macrophage-colony stimulating factor (GM-CSF) to the culture media. The authors proved that it had some protective effect on culture-induced embryo stress, therefore decreasing the miscarriage rates. There was, however, no effect on birth weight and abnormalities in comparison to the control group [13].

## 3. Does the Type of Culture Medium Affect Birth Weight of Children Born after IVF?

The research groups of Dumoulin et al. and Nelissen et al. were the first to have documented the effect of culture media on the birth weight of IVF singletons in humans. The authors agree that culture media influence perinatal outcome of IVF singletons and twins, with the exception of gestational age. Nelissen et al. compared two commercially available culture media and concluded that in vitro culture of embryos in media made by Cook Corporation resulted in singletons with a lower mean birth weight (adjusted mean difference 112 g) and more singletons with low birth weight (LBW, <2500 g) when compared to singletons born after culture media produced by Vitrolife [14]. These results are consistent with the previous findings from Dumoulin et al. based on IVF treatment cycles with transfer of fresh embryos [15]. Kleijkers et al. investigated the long-term consequences of different culture media and showed that the effect might persist long after birth. In vitro culture in Cook media resulted in smaller babies in comparison to Vitrolife culture and the differences in weight were significant even after 2 years of life [16]. In 2015 the same group of researchers compared two different culture media (G5 medium and HTF (human tubal fluid)) with regard to gene expression changes. They showed that there were significant differences in gene expression of over 900 genes between the studied media. Their results favored G5 medium, as the majority of upregulated genes were responsible for cell cycle and DNA replications and the embryos developed better [17].

Nevertheless, a few studies with somewhat conflicting results regarding growth patterns have been published since 2010. Several studies comparing cultures in G1.3/Global/G1.5, HTF/Sage, G5tm/Global/Quinn's advantage, and Medicult/Cook/Vitrolife media found no significant differences in mean birth weight between singletons cultured in those media [18–21]. One of the most recent Dutch publications by Zandstra et al. is a very detailed review on the type of culture media and their influence on human neonatal birth weight. Out of eleven identified studies five showed significant differences in birth weights between analyzed culture media, while the remaining six did not support that thesis. It only shows how difficult it is to prove the effect of early embryo development conditions on future perinatal results [22].

In contrast to Dutch studies, Belgian group of De Vos et al. published a large retrospective study which does not support earlier concerns that both the type of culture medium and the duration of embryo culture affect birth weight of IVF singletons. The authors analyzed 2098 singleton live births derived from culture in sequential media by Medicult and Vitrolife and they showed no significant differences in neonatal birth weights between two culture media. They also claim that continuous surveillance of human embryo culture procedures should remain a priority of IVF centers [23].

There are also papers examining if the source of proteins in embryo culture media can influence neonatal birth weights of IVF singletons. Zhu et al. proved that such a source might be an independent factor affecting birth weight based on multiple regression analysis [24]. Answering the question of whether or not there is a true relationship between culture conditions and perinatal outcome is extremely relevant, because elucidating these mechanisms could be helpful in reducing perinatal risks, such as low birth weight. All the above suggests that a possible impact on intrauterine growth might be related to specific culture media formulations and not to all media, but this still needs confirmation in well-powered randomized controlled trials.

Several animal studies have been performed to address the same issue. Different culture conditions, mainly achieved by the addition of serum, have led to an increased birth weight in sheep and cattle and a decreased birth weight in mice. An explanation for these culture medium-induced effects could be that in vitro culture leads to epigenetic disturbances, possibly in more fragile mitochondrial DNA particles, which in turn might affect developmental programming of fetal and placental tissues. Preimplantation embryo culture has been shown to affect methylation and expression of imprinted genes in several animal models, as mentioned above [25–28].

It seems that there is still lack of knowledge in this aspect of assisted reproductive technology. Unfortunately, the complete composition of commercially available media is unknown, so it makes it harder to conclude and speculate as to which components might be more beneficial or deleterious for development and competence. It is obvious that further studies are definitely required to make in vitro fertilization procedure a safe and responsible choice for patients.

## 4. Blastocyst versus Cleavage Stage Transfer in IVF: Are There Differences in Neonatal Outcome?

Several studies have evaluated the outcome of singleton pregnancies after blastocyst versus cleavage stage embryo transfer. Higher incidences of preterm birth (PTB), very preterm birth, low birth weight (LBW), and congenital malformations were identified in a few of them. The systematic literature review of Dar et al. compared 9506 births after day 3 transfer with 3206 babies born after blastocyst transfer. The authors concluded that embryo transfer after prolonged culture to the blastocyst stage was associated with higher odds of preterm birth compared to cleavage stage transfer. In order to explain the above they suggested that longer in vitro culture might have a deleterious effect on subsequent placentation [29].

A large Swedish study by Källén et al. examining neonatal outcomes after cleavage versus blastocyst transfer also indicated a significantly higher likelihood of preterm birth after blastocyst transfer. The authors compared 1311 infants born after blastocyst transfer versus 12562 babies after cleavage stage transfer and attributed PTB to the possibly longer period of culture [30].

In contrary to the latter study, Fernando et al. in a large cohort of 4202 women who conceived after IVF and IVF-ICSI found no differences in any of the perinatal outcomes when births resulting from transfer on days 5 to 6 were compared to transfers on days 2 to 4 [31].

The systematic review and meta-analysis by Maheshwari et al., based on the data from observational studies, found an increased risk of preterm delivery, but not small-for-gestational-age babies, in pregnancies after blastocyst transfer compared to those after cleavage stage transfer. The authors are very critical to the obtained results and suggest combining data from all existing randomized trials in an individual patient data meta-analysis to provide enough power to determine any differences when designing further studies. Although individual patient data from various registries will have higher power and can be adjusted for important confounders, one has to remember that the patients who are eligible for extended culture have very different prognosis compared with those who do not fulfill the criteria for blastocyst transfer [32].

Future research should be aimed at improving both the safety of culture media and incubation systems used for prolonged culture to the blastocyst stage.

## 5. Placental Weight in IVF Pregnancies: Could It Be of Importance for the Perinatal Outcome?

Only few studies have examined placental size as a perinatal outcome in pregnancies achieved by in vitro fertilization. An extensive analysis in the Norwegian population compared placental and newborn weights in 536 567 singleton pregnancies, including 4557 conceived by IVF, 3192 through ICSI, and 88 pregnancies by the combined use of IVF and ICSI or other unspecified ART (assisted reproductive technologies) procedures. As previously reported, ART singletons had significantly lower birth weights than spontaneously conceived controls but were additionally characterized by significantly bigger placentas. The changes in birth weight, placental size, and fetal: placental weight ratio remained significant regardless of the form of fertilization even when adjusted for length of gestation, sex, parity, maternal age, and pregnancy complications [33]. It all could be due to culture media used but definitely requires further studies. Some suggest that increased placental weight/birth weight ratio might be one of the causes of adverse perinatal outcome in IVF pregnancies.

## 6. In Vitro Maturation of Oocytes and Its Probable Effect on Perinatal Outcome

In vitro maturation (IVM) is an emerging type of ART, in which immature as opposed to mature oocytes are harvested from the ovaries and allowed to mature in vitro prior to fertilization by either standard IVF or intracytoplasmic sperm injection (ICSI). Since the first report of a human birth resulting from IVM in 1991 it is estimated that over 1,300 babies have been born following this technique, in contrast to estimated 4 million babies conceived by standard IVF and ICSI over the last 30 years. In vitro maturation, or the maturation of prophase I oocytes to metaphase II in culture dish prior to fertilization, is a newer technology with limited follow-up on childhood outcomes [34–36].

The largest and best study designed to investigate the outcomes of IVM children was published by Buckett et al. from McGill University in Montréal. The authors examined obstetric outcomes and congenital abnormalities in pregnancies conceived after IVM (55 cases), IVF, and ICSI and compared with those of spontaneously conceived controls. They concluded that while all ART pregnancies were associated with an increased risk of multiple pregnancy, cesarean delivery, and congenital abnormalities, IVM was not associated with any additional risk compared with IVF or ICSI. Interestingly, the mean birth weight of IVM infants was higher than in controls, IVF, and ICSI infants, which may be suggestive of epigenetic differences in IVM children. Different culture media used in this particular procedure should also be taken into account [37].

Further analyses of perinatal outcome of babies born after IVM are required, as this method has emerging promises for patients at risk of fertility loss due to emergency gynecological surgery or oncological states requiring fertility preservation.

## 7. Does Cryopreservation of Embryos Affect the Perinatal Outcome?

Constantly improving cryopreservation techniques are a guarantee for future success of ART. Cryopreservation and thawing processes can uniquely affect pregnancy outcomes. The available literature regarding that subject is generally in favor of frozen/thawed embryo transfers. Singleton pregnancies after transfer of frozen/thawed embryos are associated with a lower risk of perinatal mortality, small-for-gestational-age babies, preterm birth (<37 weeks), low birth weight (<2500 g), and antepartum hemorrhage when compared with those after fresh embryo transfers. They are also at increased risk of macrosomia or being large for gestational age [38, 39]. A systematic review and meta-analysis of Maheshwari et al. regarding frozen-thawed embryo transfers included 11 studies. The authors proved that singleton pregnancies derived from thawed transferred embryos were associated with better perinatal outcome. The risk of preterm delivery was significantly reduced in comparison with fresh transfers (RR 0.84, 95% CI 0.78–0.90), so was the risk of low birth weight (RR = 0.69, 95% CI 0.62–0.76) and perinatal mortality (RR = 0.68, 95% CI 0.48–0.96) [40]. It must be remembered however that the literature discusses both types of cryopreservation (slow freezing and vitrification). It is well known that the pregnancy rates after vitrification are better than those following slow freezing and thawing. Nevertheless, once the embryo survives the thawing process and implants, the difference in perinatal outcome is not known. It is however thought to be related to maternal hormonal environment in the stimulated and natural cycles more than to the effect of the technique of freezing/thawing itself.

## 8. Conclusions

Culture media are one of the puzzles pieces in the success of IVF. Other factors such as incubator gases, temperature, culture oils, and supplements are equally important and can change the dynamics of how the embryo interacts with components of the medium. Characteristics of other parameters, such as pH and oxygen concentration, can also affect the embryos and should be considered together when analyzing the overall result of IVF. The implementation of new technologies brings us closer to robotic IVF, but we still need to provide the early human being with the conditions mimicking the female reproductive tract. Therefore, culture media will always play a major role in the IVF laboratory. The variety of factors affecting the results such as adverse perinatal outcome, neonatal birth weight, placental weight, and epigenetic changes makes it very hard—almost impossible—to draw conclusions. This results in many papers showing conflicting data. Hopefully, unification of products and clear indications for the procedure will allow the performance of prospective, randomized studies with satisfactory properly powered results and conclusions.

Regarding the obstetric and perinatal outcome, it seems that “freeze-all” strategy may one day dominate IVF procedures, as there is increasing evidence of favorable outcome in such cycles.
